# Determinants of Right Ventricular Performance in Severe Acute Pulmonary Embolism

**DOI:** 10.1016/j.jscai.2025.103868

**Published:** 2025-08-19

**Authors:** Zachary D. Demertzis, Terry R. Bowers, James A. Goldstein

**Affiliations:** Department of Cardiovascular Medicine, Corewell Health William Beaumont University Hospital, Royal Oak, Michigan

**Keywords:** echocardiography, interventricular septum, pulmonary embolism, right ventricle, right ventricular dysfunction

## Abstract

**Background:**

Severe acute pulmonary embolism (PE) induces hemodynamic compromise due to a failing right ventricle (RV) and a “dry” hyperdynamic left ventricle (LV). RV systolic dysfunction is the key parameter to determine acute PE risk stratification, clinical management, and prognosis. The present study delineates the determinants of RV performance in acute PE resulting in RV dysfunction.

**Methods:**

This was a single-center, retrospective analysis of a high-volume PE response team database of patients with intermediate-high-risk or high-risk PE with an echocardiogram prior to escalation of care.

**Results:**

The RV free wall motion (total RVFW motion score = 8.1 ± 2.8) was correlated with the magnitude of RV systolic depression (RV fractional area change [FAC] = 29 ± 13%, tricuspid annular planar systolic excursion = 1.57 ± 0.49 cm, and S′ velocity = 10.57 ± 3.14 cm/s). LV preload and stroke volume were markedly reduced (LV end diastolic size = 4.04 ± 0.68 cm and volume = 73.6 ± 25.8 mL; LV stroke volume = 46.2 ± 16.6 mL). LV preload deprivation was correlated with the severity of RV systolic dysfunction (total RVFW motion score, *r* = −0.11, *P* = .39; FAC, *r* = 0.25, *P* = .04; S′ velocity, *r* = 0.27, *P* = .03). RV overload induced reversed interventricular septal curvature reflected by LV end diastolic eccentricity index = 1.21 ± 0.21, which correlated with RV systolic dysfunction (total RVFW motion score, *r* = 0.47, *P* < .001; FAC, *r* = −0.35, *P* < .005; S′ velocity, *r* = −0.43, *P* < .001) and RV dilation.

**Conclusions:**

Afterload strain imposed by PE may induce severe RV systolic dysfunction attributable to marked RVFW dysfunction. RV systolic pressure generation and transpulmonary flow are generated through systolic ventricular interactions mediated by primary septal contraction and paradoxical septal motion.

## Introduction

Severe acute pulmonary embolism (PE) induces hemodynamic compromise characterized by low cardiac output (CO) and hypotension, a result of conditions in which the right ventricle (RV) is failing and dilated (“strained”), whereas the left ventricle (LV) is “dry” but contracting vigorously.^1^ Low CO results from LV preload deprivation, a consequence of thrombus-induced pulmonary arterial obstruction that directly diminishes transpulmonary flow from RV to LV.^2^^,^^3^ Reduced LV filling is exacerbated by acute RV systolic dysfunction attributable to acute afterload stress imposed by elevated pulmonary vascular resistance upon the “unprepared” thin-walled RV, which further impairs transpulmonary flow.^4^

RV systolic dysfunction is the key parameter upon which acute PE risk stratification, clinical management, and prognosis are based.^5^, ^6^, ^7^ PE impact is categorized by the presence of RV “strain” by computed tomography angiogram (CTA)-documented RV dilatation (RV:LV size >1.0).^7^^,^^8^ However, “RV size” is merely a surrogate indicator of RV systolic dysfunction, lacking capability to characterize the magnitude of impaired RV free wall (RVFW) contraction and depressed global RV pump performance. Importantly, previous studies of PE-induced RV systolic dysfunction have not elucidated the mechanisms by which RV systolic performance and transpulmonary flow are generated when the RV is profoundly depressed. The present study was designed to delineate the determinants of RV performance in acute PE resulting in RV dysfunction.

## Materials and methods

This single-center retrospective study analyzed a high-volume PE response team (PERT) database, comprising 3964 consecutive patients from 2020-2023 who had undergone echocardiography prior to escalation of care (EOC). Cases escalated to therapy (mechanical aspiration thrombectomy or thrombolytic therapy) were categorized as either intermediate-high-risk (RV strain [RV:LV ratio ≥1.0 by CTA] together with elevated biomarkers [troponin, brain natriuretic peptide] but intact systolic blood pressure) or high-risk (similar imaging and biomarker factors with the addition of hypotension).

### Risk stratifications and treatment recommendations

The PERT process is implemented as follows: (1) the rapid response team (24 hours/7 days per week) evaluates every patient 18 years and older with confirmed PE by CTA; and (2) multidisciplinary PERT call (cardiology fellow, interventional cardiologist, and interventional radiologist) is rapidly convened for virtual case review. Recommendations for EOC are based on severity of RV strain, clinical hemodynamic impact (magnitude of tachycardia and hypoxemia), extent and pattern of pulmonary arterial (PA) clot burden, and other relevant clinical factors (ie, age, comorbidities, life expectancy, and bleeding risk). Simplified pulmonary embolism severity index (sPESI) score is prospectively calculated for all patients and utilized in the risk stratification and PERT multidisciplinary discussion.

### Echocardiographic analysis of RV and LV function

The echocardiograms were reviewed by 2 independent observers who assured the images were of sufficient quality to allow the analyses to be performed; images deemed of insufficient quality were excluded from the analysis. Assessment of the RV by echocardiography was performed according to established methods.^9^, ^10^, ^11^ RV systolic function was assessed by: (1) RV fractional area change, measuring the RV chamber areas at end-diastole and end-systole in the apical 4-chamber view; (2) RVFW motion score, assessed qualitatively in the basal, mid, and apical segments (1 = normal, 2 = hypokinetic, 3 = akinetic, and 4 = dyskinetic), with the cumulative sum score of the 3 segments used to calculate a total RVFW motion score; and (3) tricuspid annular planar systolic excursion and S′ velocities, measured according to standard methods. RV diastolic function was measured as RV end diastolic size. Under conditions of severe RVFW dysfunction induced by RV infarction (RVI), RV performance was determined by LV-septal contractile contributions mediated by paradoxical systolic septal motion.^12^ To assess whether such compensatory patterns were present in acute PE, interventricular septal (IVS) motion was analyzed qualitatively by tracking directional changes frame-by-frame from end-diastole to end-systole in the short axis view.

The LV systolic function was assessed by LV ejection fraction and stroke volume by biplane volume measurement.^10^ To assess LV preload deprivation, the LV internal diameter at end diastolic size and LV end diastolic volume by biplane measurement were employed. Further, to assess the impact of RV dilation and dysfunction on diastolic ventricular interactions, IVS contour was measured as an eccentricity index in the parasternal short axis as previously described.^13^ The relationships of IVS diastolic eccentricity and systolic motion patterns were compared to the extent of RV systolic dysfunction, including both RV FAC and RVFW motion score. The extent of LV preload deprivation measured by LV diastolic size was similarly compared to measures of RV dysfunction. To assess the relationship between the RV and the pulmonary arterial circuitry (RV-PA coupling), the ratio of RV fractional area change to RVSP determined by echocardiography was used.

### Statistical analysis

Mean ± SD are presented for continuous variables. Absolute frequencies are reported for categorical variables. At baseline, missing data for these components were infrequent (<1%), except for tricuspid regurgitation velocities and right ventricular systolic pressure measurements (approximately 33%) due to trace or no tricuspid regurgitation. Given the low rate of missing data, our analyses primarily relied on observed data unless explicitly explained otherwise. A Pearson correlation coefficient was used to assess the strength and association between numerical variables.

## Results

### Demographics

Of a total population of 3964 patients with intermediate-high- or high-risk PE, N = 66 had an echocardiogram completed before EOC (91% intermediate-high-risk and 9% high-risk). Patient demographic characteristic data and clinical characteristics are summarized in Table 1. The majority of patients (73%) underwent mechanical thrombectomy.

**Table 1:** Patient demographic characteristics, baseline hemodynamics, cardiac biomarkers, and CT measurements.

Characteristic	N = 66
Sex	
Male	34 (52%)
Female	32 (48%)
Body mass index, kg/m^2^	33.0 ± 7.6
Age, y	64 ± 13.4
Therapy	
Mechanical thrombectomy	48 (73%)
Thrombolytic therapy	18 (27%)
Heart rate, bpm	98 ± 20.0
Blood pressure, mm Hg	
Systolic	127 ± 29.5
Diastolic	77 ± 14.0
Hypotension	1 (2%)
Respiratory rate	22 ± 5.5
Oxygen saturation, %	95 ± 4
Troponin I, ng/mL	0.42 ± 0.68
Brain natriuretic peptide, pg/mL	365.6 ± 451.18
CT LV:RV ratio	1.54 ± 0.55
sPESI	2.0 ± 1.2
Risk stratification	
Intermediate-high	60 (91%)
High	6 (9%)
PA catheterization, mm Hg	
PASP	49.6 ± 15.3
Mean PA pressure	27.3 ± 8.8

Values are mean ± SD or n (%).

CT, computed tomography; LV, left ventricle; PA, pulmonary artery; PASP, pulmonary artery systolic pressure; RV, right ventricle; sPESI, simplified pulmonary embolism severity index.

### Baseline hemodynamic and imaging characteristics

Baseline hemodynamics, cardiac biomarkers, and imaging parameters are summarized in Table 1. Patients manifested significant RV enlargement (RV:LV ratio of 1.54 ± 0.55 by computed tomography) but were hemodynamically stable (aortic systolic pressure 127 ± 29.5 mm Hg), but given the evidence of marked RV strain, they not surprisingly manifested elevated biomarkers (troponin I 0.42 ± 0.68 ng/mL and brain natriuretic peptide 365.6 ± 451.18 pg/mL). Prior to thrombectomy, pulmonary hypertension was evident with invasive PA systolic pressure elevated to peak 49.6 with mean 27 mm Hg. The average sPESI score was 2 ± 1.2.

**Figure 1:**
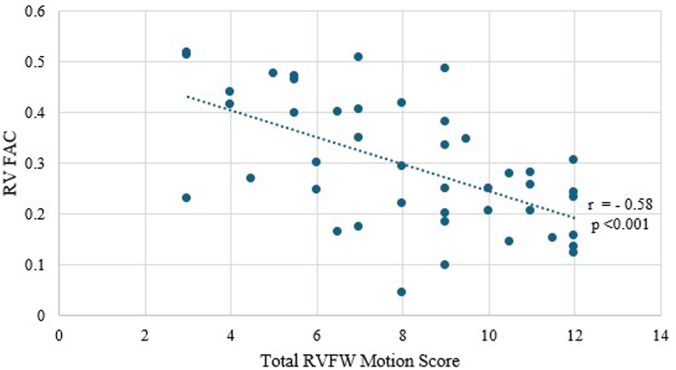
**Correlation of total RVFW motion score and RV FAC.** FAC, fractional area change; RV, right ventricle; RVFW, right ventricular free wall.

Echocardiographic parameters are summarized in Table 2. As expected, based on the patient cohort, patients manifested severe RV dysfunction. There was marked impairment of RVFW motion (basal wall = 2.9 ± 1.1, mid wall = 3.3 ± 1.0, and apical wall = 2.0 ± 1.1; total RVFW motion score = 8.1 ± 2.8), which correlated with the magnitude of global RV systolic depression (RV FAC = 29 ± 13%, reduced tricuspid annular planar systolic excursion = 1.57 ± 0.49 cm, and S′ velocity = 10.57 ± 3.14 cm/s); Figure 1). Of note, despite severe RV enlargement, significant tricuspid regurgitation was uncommon (trace or less in 65% of cases). There was RV-PA uncoupling with an FAC/RVSP ratio of 0.55 ± 0.31%/mm Hg.

**Table 2:** Echocardiographic measurements.

Measurement	N = 66
Ejection fraction, %	59 ± 9
LVIDd, cm	4.04 ± 0.68
LVIDs, cm	2.57 ± 0.57
LV end systolic volume, mL	28 ± 12.7
LV end diastolic volume, mL	73.6 ± 25.8
Stroke volume, mL	46.2 ± 16.6
RV:LV ratio	1.16 ± 0.30
PASP, mm Hg	47.7 ± 11.8
Right atrial pressure, mm Hg	11.2 ± 4.3
Pear tricuspid regurgitation velocity, m/s	3.02 ± 0.61
Tricuspid regurgitation severity	
None	14 (21%)
Trace	29 (44%)
Mild	13 (20%)
Moderate	7 (11%)
Severe	3 (5%)
RV end diastolic area, cm^2^	24.61 ± 6.32
RV end systolic area, cm^2^	17.37 ± 5.64
RV FAC	29 ± 13
TAPSE, cm	1.57 ± 0.49
S′, cm/s	10.57 ± 3.14
RV free wall motion score	8.1 ± 2.8
Basal	2.9 ± 1.1
Mid	3.3 ± 1.0
Apical	2.0 ± 1.1
RV FAC/RVSP, %/mm Hg	0.55 ± 0.31
End diastolic eccentricity index	1.21 ± 0.21

Values are mean ± SD or n (%).

FAC, fractional area change; LV, left ventricle; LVIDd, left ventricular internal diameter at end-diastole; LVIDs, left ventricular internal diameter at end-diastole end-systole; PASP, pulmonary artery systolic pressure; RV, right ventricle; RVSP, right ventricular systolic pressure; TAPSE, tricuspid annular planar systolic excursion.

LV ejection fraction was preserved (59% ± 9%), but LV preload was markedly reduced (LV end diastolic size = 4.04 ± 0.68 cm and volume = 73.6 ± 25.8 mL), associated with reduced LV stroke volume (46.2 ± 16.6 mL). LV preload deprivation correlated with the severity of RV systolic dysfunction (total RVFW motion score, *r* = −0.11, *P* = .39; FAC, *r* = 0.25, *P* = .04; S′ velocity, *r* = 0.27, *P* = .03). RV overload induced reversed IVS curvature reflected by LV end diastolic eccentricity index = 1.21 ± 0.21, which correlated with both RV systolic dysfunction (total RVFW motion score, *r* = 0.47, *P* < .001; FAC, *r* = −0.35, *P* < .005; S′ velocity, *r* = −0.43, *P* < .001) and RV dilation.

## Discussion

Hemodynamically severe PE is characterized by low CO due to reduced LV preload delivery, the proximate cause being thrombotic obstruction directly impeding transpulmonary flow. Abrupt PE-induced elevated pulmonary vascular resistance precipitates severe RV dysfunction, which exacerbates LV underfilling due to depressed RV ejection fraction. The magnitude of RV dysfunction is associated with more severe hemodynamic compromise and imparts worse prognosis.

Observations from the present study delineate the pathophysiologic derangements that characterize RV systolic dysfunction and the determinants of RV performance under conditions of acute PE. These findings document that PE imposed severe afterload strain induces marked RVFW dysfunction, often rendering it akinetic to dyskinetic. Despite lack of visible RVFW contraction, RV systolic pressure and transpulmonary flow are actively generated through systolic ventricular interactions mediated by primary septal contraction and paradoxical septal motion (Central Illustration).

**Central Illustration:**
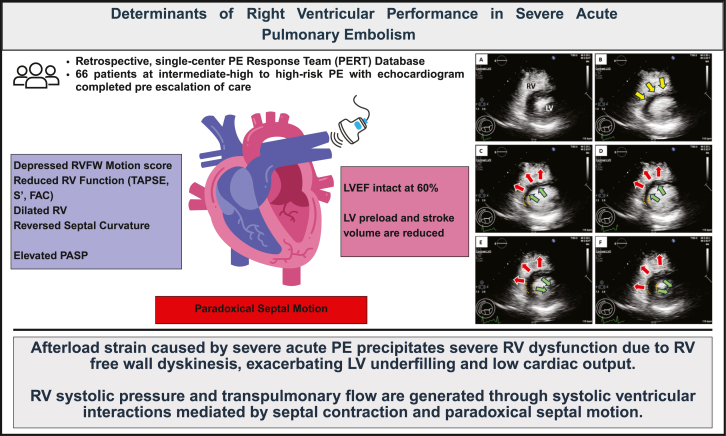
**Determinants of right ventricular performance in severe acute PE.** FAC, fractional area change; LV, left ventricular; LVEF, left ventricular ejection fraction; PASP, pulmonary artery systolic pressure; PE, pulmonary embolism; RV, right ventricular; RVFW, right ventricular free wall; TAPSE, tricuspid annular planar systolic excursion.

### RV architecture and mechanics

**Figure 2:**
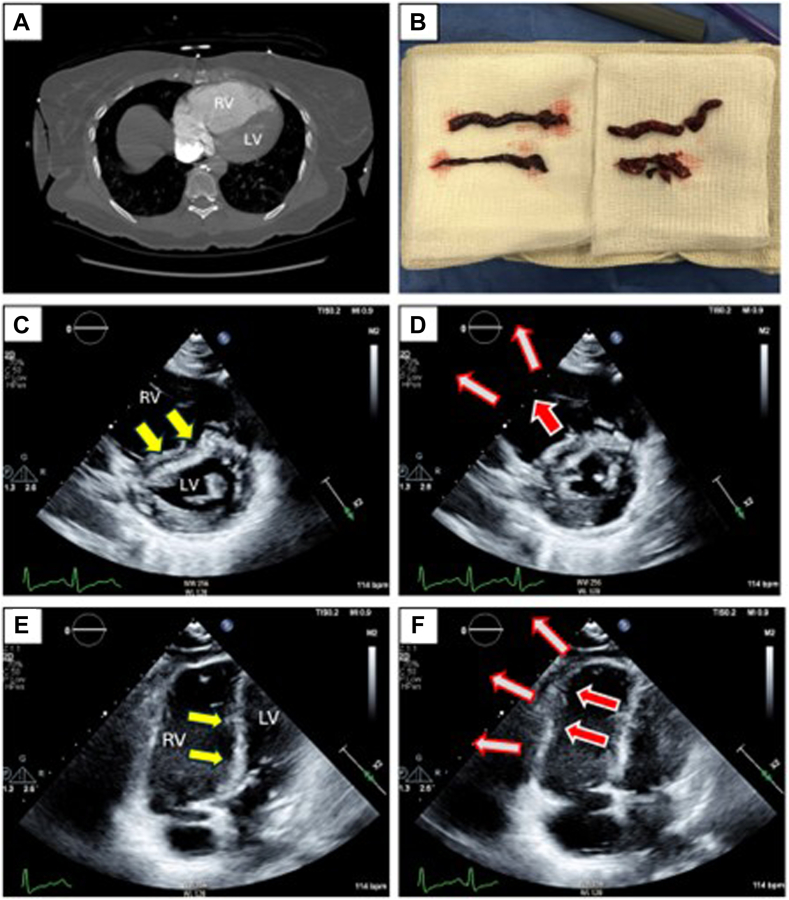
**Patient with high-risk pulmonary embolism and sPESI 3.** Prethrombectomy echocardiographic contrast images illustrating frame-by-frame systolic IVS motion pattern. (A) End-systole frame. (B) At end-diastole, the RV is dilated and the IVS is flattened (yellow arrows) toward the LV. (C) In early systole, the IVS moves paradoxically, with both right side (red arrows) and left side (green arrows) moving toward the RV. (D) As systole progresses, both IVS surfaces move further into the RV. (E) As IVS thickening and LV shortening ensue, the 2 sides of the septum move in opposite directions and the left IVS surface is pulled toward the LV lateral wall (green arrows), while the right IVS surface continues to move paradoxically into the RV (red arrows). (F) At end-systole, the IVS is fully thickened, and the discordant motions of the right and left surfaces are further exaggerated. LV, left ventricle; IVS, interventricular septum; RV, right ventricle; sPESI, simplified pulmonary embolism severity index.

An appreciation of RV structure and function is essential to the pathophysiologic derangements and compensations for RV dysfunction in hemodynamically severe PE. The RV is a pyramidal shaped thin-walled volume pump, with its myocardium constructed in a 3-dimensional network of myofibers arranged in a multihelical pattern.^14^ The RVFW is a wrap-around circumferential muscle, with the epicardial layer constituting 25% of the wall. It spans the LV and encompasses the subpulmonary infundibulum, and it courses parallel with the atrioventricular groove, spiraling at the apex into the predominant subendocardial longitudinal layer, which is continuous with the IVS. The IVS helical fibers are oblique and cross each other at 60° angles, comprising components of the helical ventricular myocardial band responsible for the radial “bellows” motion generating 20% of RV output. Oblique subendocardial fibers produce longitudinal shortening, with traction of the tricuspid annulus toward the apex responsible for 80% of RV systolic function.^15^^,^^16^ The IVS is anatomically and functionally bilayered, constituting an integral architectural and mechanical element of the RV. Echocardiographic speckle tracking and 3-dimensional studies have illustrated distinct RV and LV architectural and mechanical IVS components, delineated as a hyperechoic line with disparate motion patterns as the right side bulges toward the RV cavity, with opposite directional shortenings in longitudinal strain toward the LV (Figure 2).^17^ Even under physiologic conditions, the IVS generates one-third of global RV stroke work through longitudinal shortening and contributions of RV septal helical fibers.^18^^,^^19^

### Determinants of RV systolic performance in hemodynamically severe PE

**Figure 3:**
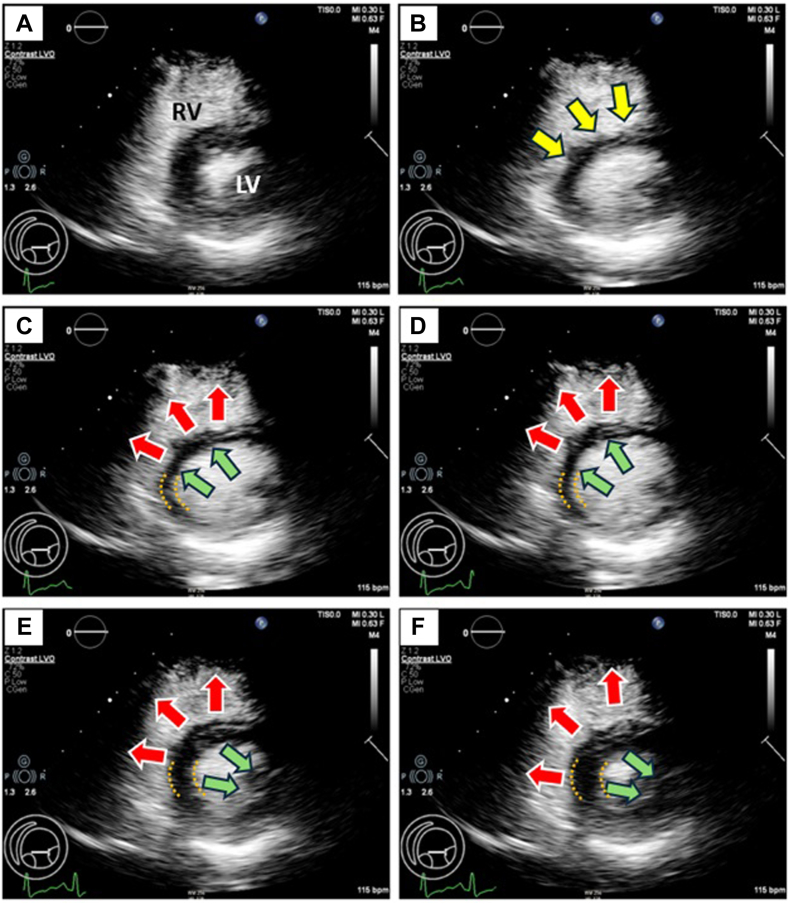
**Patient with high-risk pulmonary embolism, with sPESI 3.** (A) Computed tomography angiogram shows right ventricle (RV) greater than left ventricle (LV) size; although not illustrated, bilateral proximal pulmonary artery thrombi were present. (B) Extracted extensive thrombus post bilateral aspiration thrombectomy. (C-F) Prethrombectomy echocardiographic images at end-diastole in short axis (C), and apical 4-chamber views (E) show RV dilation and reversed curvature of the interventricular septum (IVS) (yellow arrows) toward the volume-deprived LV. In systole (D, F), the right ventricular free wall was dyskinetic (open red arrows), with the IVS bulging paradoxically into the RV (closed red arrows). sPESI, simplified pulmonary embolism severity index.

The present findings delineate the patterns of RV dysfunction due to acute PE and the mechanisms by which transpulmonary flow is generated when the RV is profoundly depressed. These echocardiographic observations document that global RV systolic dysfunction is attributable to markedly depressed RVFW contraction, with segments commonly akinetic and in some cases frankly dyskinetic, the severity of regional wall motion abnormalities correlated with the magnitude of depressed RV systolic function and associated with reduced LV filling and stroke volume. These results document for the first time the mechanisms by which RV output is generated under conditions of PE-induced severely impaired RVFW motion. Despite lack of visible RVFW contraction, an active PA systolic waveform and transpulmonary flow were generated by LV-septal contractile contributions through systolic ventricular interactions mediated by primary septal contraction and paradoxical septal motion (Figure 3). Given that a significant component of RV pump function measured by RV FAC was generated by LV-septal contractile contributions, the true impact of depressed RVFW contractions on depressed global RV systolic performance (Figure 1) was likely underestimated.

Hemodynamic compromise in acute PE and the compensatory mechanisms responsible for generating RV systolic performance under conditions of severe RVFW dysfunction are informed by lessons from acute RVI, which shares in common the hemodynamic signature of low CO-hypotension in the setting of disparate RV-LV conditions in which the RV is depressed and dilated and the LV “dry” with intact contraction.^20^ Prior studies of RVI in experimental animals and humans similarly demonstrate that acute RV dysfunction due to ischemia reduces transpulmonary delivery of LV preload, resulting in low CO-hypotension despite intact LV systolic function.^12^^,^^21^ Consistent with the present findings in acute PE, with the RVFW ischemically impaired, RV systolic performance is generated by systolic ventricular interactions mediated by primary septal contraction and paradoxical septal motion (Figure 2).

### Motion patterns of the biventricular IVS

Systolic interactions were mediated by distinct and discordant systolic IVS motion patterns, inscribed as a bilayered and bimodal motion with disparate movements of right and left septal components as phases of systole progressed. In the earliest isovolumic systolic phase, there was abrupt unidirectional paradoxical bulging of the entire IVS into the RV, reflecting unopposed LV-septal pressure generation due to lack of RVFW contraction and stretching of the dyssynergic RVFW to its maximal systolic lengthening, thereby providing a buttress to facilitate the initial active RV systolic pressure generation and outflow. As fiber shortening ensued during ejection, a bilayer bidirectional pattern was manifest, wherein the left-sided septal component reversed direction contracting toward the LV lateral wall, while concomitantly the right side of the septum continued to bulge into the RV. These IVS motion patterns reflect its architectural-mechanical relationships with the LV myocardium, whereby the right and left side of the septum belong to and are mechanically influenced by distinct components of the helical myocardial fibers.

### RV-induced adverse diastolic ventricular interactions

The magnitude of RV systolic dysfunction and dilatation correlated with the extent of compromised LV filling, the proximate cause of low output. Reduced LV preload was not only the result of RV systolic dysfunction resulting in depressed transpulmonary preload delivery but was further exacerbated by adverse diastolic septal interactions induced by RV dilation and its associated pressure elevation (Figures 2 and 3). Echocardiography eccentricity indices consistently documented IVS end diastolic reverse curvature, the magnitude of which being associated with more severe RV dysfunction. Similar IVS-mediated adverse diastolic interactions are characteristic of acute RVI, in which RV diastolic dilation and pressure elevation impair filling and compliance of the contralateral LV; such interactions are intensified under conditions of intrapericardial pressure elevation, as occurs under conditions of abrupt RV dilation within the noncompliant pericardium.^12^^,^^21^ Analogous intrapericardial crowding likely occurs in acute PE-induced RV dilation, and although not measured in the present study, prior studies have documented pulsus paradoxus in both acute PE and RVI, likely a reflection of pericardial intensified adverse right-to-left diastolic interactions.^22^

### Clinical implications

The clinical importance of the afterload-strained RV is emphasized by its primacy as the key parameter upon which risk stratification, clinical decision making, and prognosis are based.^23^ It is important to emphasize that PE-induced RV dysfunction is exacerbated by RV ischemia, even in the presence of normal coronary anatomy. Severe RV afterload excess and RV dilation increase oxygen demand on the thin-walled RV, rendering it prone to ischemia. Under these conditions, the RV is disproportionately dependent on optimal aortic perfusion, in which hypotension precipitates hemodynamic decline that may propel the failing RV into a spiraling “Doom Loop.”^24^^,^^25^ It follows that restoration of aortic perfusion is paramount. The present observations may be extrapolated to provide a strong argument against the oft used intervention of volume loading, a common “therapeutic reflex” practiced in many shock states whereby administration of volume resuscitation is a first and immediate strategy. Though efficacious in hypovolemic shock, in predominant RV shock attributable to PE, volume loading the struggling dysfunctional dilated RV is unlikely to increase output through the thrombotically obstructed pulmonary vasculature. Therefore, there is no reasonable basis upon which to propose—or hope—that further distending this severely dilated chamber by “forced feeding” would improve LV preload delivery and would more likely exacerbate RV ischemia and failure. Current therapeutic regimens emphasize urgent vasopressor augmentation of mean aortic pressure while definitive interventions to relieve PA thrombus burden are initiated.^26^

### Limitations

There are important limitations pertinent to the methods of this study. First, the cohort analyzed was selected based on the availability of an echocardiographic study during the acute PE phase of sufficient quality to assess detailed RV performance, thereby constituting potential selection bias. The data showed differences in RV:LV ratios by echo (true end diastolic image) vs CTA (nongated image); whereas echo is most accurate. For EOC decision making, CT ratio is immediately available with the diagnostic CTA and is sufficient for that purpose. Echo delineation of RV dysfunction severity may be clinically useful in patients hemodynamically unstable after therapeutic intervention in whom persistent RV dysfunction may contribute to compromise and necessitate mechanical circulatory support.

The significant correlation between RVFW dysfunction and RV FAC may have been underestimated by the fact that under conditions of severe RVFW dysfunction, RV global performance is predominantly determined by IVS contraction. Further, the present investigation did not analyze the natural history of RV performance in response to therapeutic interventions; further studies will be necessary to delineate the patterns of RV recovery after successful relief of PE-induced afterload strain.

## Declaration of competing interest

The authors declared no potential conflicts of interest with respect to the research, authorship, and/or publication of this article.
